# Heterogeneity of Circulating Tumor Cells in Neoadjuvant Chemotherapy of Breast Cancer

**DOI:** 10.3390/molecules23040727

**Published:** 2018-03-22

**Authors:** Evgeniya V. Kaigorodova, Olga E. Savelieva, Liubov A. Tashireva, Natalia A. Tarabanovskaya, Elena I. Simolina, Evgeny V. Denisov, Elena M. Slonimskaya, Evgeny L. Choynzonov, Vladimir M. Perelmuter

**Affiliations:** 1 Cancer Research Institute, Tomsk National Research Medical Center, Tomsk 634050, Russia; olga_chechina@mail.ru (O.E.S.); lkleptsova@mail.ru (L.A.T.); tarabanovskaya@inbox.ru (N.A.T.); simolin12@sibmail.com (E.I.S.); d_evgeniy@oncology.tomsk.ru (E.V.D.); slonimskaya@yandex.ru (E.M.S.); nii@oncology.tomsk.ru (E.L.C.); pvm@ngs.ru (V.M.P.); 2 Siberian State Medical University, Tomsk 634050, Russia; 3 Laboratory for Translational Cellular and Molecular Biomedicine, Tomsk State University, Tomsk 634050, Russia

**Keywords:** heterogeneity of circulating tumor cells, EMT, EpCAM-negative circulating tumor cells, NACT, breast cancer

## Abstract

The biological properties of circulating tumor cells (CTCs), and their dynamics during neoadjuvant chemotherapy are important, both for disease progression prediction and therapeutic target determination, with the aim of preventing disease progression. The aim of our study was to estimate of different CTC subsets in breast cancer during the NACT (neoadjuvant chemotherapy). The prospective study includes 27 patients with invasive breast cancer, T2-4N0-3M0, aged 32 to 60 years. Venous heparinized blood samples, taken before and after biopsy, after each courses of chemotherapy (on days 3–7), and before surgical intervention, served as the material for this study. Different subsets of circulating tumor cells were determined on the basis of the expression of EpCAM, CD45, CD44, CD24, and N-Cadherin using flow cytometry. As the result of this study, it has been observed that significant changes in the quantity of the different subsets of circulating tumor cells in patients’ blood were observed after carrying out the 3rd course of NACT. NACT causes significant changes in the quantity of six CTC subsets, with various combinations of stemness and epithelial-mesenchymal transition (EMT) properties.

## 1. Introduction

Apparently, the presence of CTCs (circulating tumor cells) is not always followed by metastases, as not all tumor cells appearing in the bloodstream have the necessary properties. CTCs are a heterogeneous population. Some cells are cancer stem cells, other cells are in an EMT (epithelial-mesenchymal transition) state, and most of the cells do not have EMT and stemness properties [1,2,3]. A meta-analysis of 24 studies (3701 patients), including 13 prospective and 11 retrospective studies devoted to the determination of prognostic importance of CTC detection on patients with breast cancer, shows that a large number of CTCs is associated with poor treatment response, shortening of the overall and disease-free survival rates [4]. It was shown that neoadjuvant chemotherapy (NACT) on breast cancer does not act on CTCs in the EMT state [5]. Nevertheless, existing studies do not have complete information about which CTC subsets are associated with haematogenous metastases and poor outcome, and how different types of CTCs react to NACT.

Therefore, the aim of our study is to estimate different CTC subsets in breast cancer during the NACT.

## 2. Results and Discussion

On the basis of the determination of the membrane expression of EpCAM, CD44, CD24, CD45, and N-Cadherin, in our study, we determined six subsets of CTCs: CTC-1—circulating tumor cells without stemness and EMT properties (EpCAM+CD45-CD44-CD24-Ncadherin-); CTC-2—circulating tumor cells without stemness and with EMT properties (EpCAM+CD45-CD44-CD24-Ncadherin+); CTC-3—circulating tumor stem cells without EMT properties (EpCAM+CD45-CD44+CD24-Ncadherin-); CTC-4—circulating tumor stem cells with EMT properties (EpCAM+CD45-CD44+CD24-Ncadherin+); CTC-5—circulating tumor stem cells with an absence of EpCAM membrane expression and without EMT properties (EpCAM-CD45-CD44+CD24-Ncadherin-); and CTC-6—circulating tumor stem cells with an absence of EpCAM membrane expression and with EMT properties (EpCAM-CD45-CD44+CD24-Ncadherin+).

Breast cancer patients show significant variability in terms of the quantity of the studied CTC subsets in peripheral blood before diagnostic and treatment procedures. Thus, CTC-3 was detected in minimal concentrations—0.00 (0.00–0.43) cells per μL. In comparison, the concentrations of CTC-1 and CTC-5 are significantly higher (respectively, 0.546 (0.00–1.11) cells per μL (*p* = 0.001) and 0.18 (0.00–2.42) cells per μL (*p* = 0.011)) (Table 1). All CTC subsets were absent in the peripheral blood of healthy donors (0.00 (0.00–0.00) cells per μL).

In a previously published study, we showed that, after biopsy of invasive breast cancer, the quantity of CTC-1 without stemness and EMT properties (EpCAM+CD45-CD44-CD24-Ncadherin-) and CTC-3 with stemness and without EMT properties (EpCAM+CD45-CD44+CD24-Ncadherin-) were increased [6].

Neoadjuvant chemotherapy was carried out after biopsy, which, itself, affects CTC quantity. Therefore, the quantity of each CTC subsets after a regular chemotherapy course was compared with the quantity of respective CTC subsets after biopsy. As the result of this prospective study, the most significant changes in the levels of different CTC subsets were observed during the first three courses of NACT. The total number of CTCs tended to increase after the 3rd course of NACT and significantly increased before the surgery regardless of the presence or absence of stem or EMT features. Various CTC subsets reacted to NACT differently. The only CTC subset with no changes after NACT was CTC-2 (cancer cells without stemness and with EMT properties) (Table 2).

Despite the fact that initial quantity of CTCs without stemness and EMT properties (CTC-1) was large and increased after biopsy, the number of these cells grew after NACT. The tendency of growth appeared after the first course (*p* = 0.07), while significant growth of that cell’s quantity was registered after the second course (*p* = 0.027), as well as after the end of NACT and before surgical treatment (*p* = 0.046) compared with the level of these cells in patient blood after biopsy. It should be noted that the presence of stemness properties in four CTC subsets does not guarantee an equal response to NACT. The quantity of stem EpCAM+ CTC-3 and EpCAM- CTC-5 without EMT properties after the third course of NACT increased on the level of a trend (*p* = 0.101 and *p* = 0.068, respectively). The quantity of stem CTC-4 cells with membrane expression of EpCAM and EMT properties decreased, and the level of these cells in blood after NACT (before surgical treatment) was significantly lower compared with the same characteristics after biopsy, and equaled 0.01 (0.00–0.71) cells per μL and 0.219 (0.00–0.55) cells per μL, respectively (Z = 2.02, *p* = 0.043).

Unlike other stem CTC subsets, after three courses of NACT, a heavy increase in the quantity of CTC-6 (with an absence of EpCAM membrane expression and with EMT properties) to 2.95 (1.25–13.28) cells per μL, compared with the level of these cells in blood after biopsy (0.16 (0.04–1.59) cells per μL, Z = 2.19, *p* = 0.027) was observed in patient blood.

The results of the study showed that NACT has a significant impact on CTC quantity. It is possible to say that CTC subsets are sensitive to NACT, to a greater or lesser degree. Studied CTC subsets were characterized using various combination of EpCAM membrane expression, stem markers (CD44+CD24-), and EMT markers (N-Cadherin+). It seems that the ability of tumor cells to express or not express the mentioned molecules does not play a fundamental role in their capacity to intravasate. CTC levels changes by many mechanisms: Cells’ ability to intravasate, loss of epithelial markers (such as EpCAM and cytokeratines), extravasation, and the intravascular death of tumor cells. Considering that the half-life of CTCs is very short, and is equal to 1–2.5 h [7], determination of the formation mechanisms of CTCs with different phenotypes will always be a cause of difficulties.

EpCAM is a main epithelial antigen for CTCs isolation and characterization. Thus, CTCs with a loss of EpCAM membrane expression can’t be detected [8,9,10]. Therefore, cells with an absence of EpCAM membrane expression are beyond the interest of scientists. EpCAM (Epithelial cell adhesion molecule) is a molecule of cell adhesion and a transmembrane glycoprotein expressing only on the epithelium [11]. EpCAM is involved, not only in cell adhesion, but also in signaling [12,13,14], proliferation, differentiation and migration [15,16]. Numerous immunohystochemical studies have shown that EpCAM overexpresses in the cells of breast, prostatic, ovarian, lungs, colon, renal and gastric carcinoma [17,18,19]. A decrease of EpCAM expression is associated with epithelial-mesenchymal transition. In addition, there is some evidence concerning the reduction of EpCAM expression in CTCs [20].

**Figure 1:**
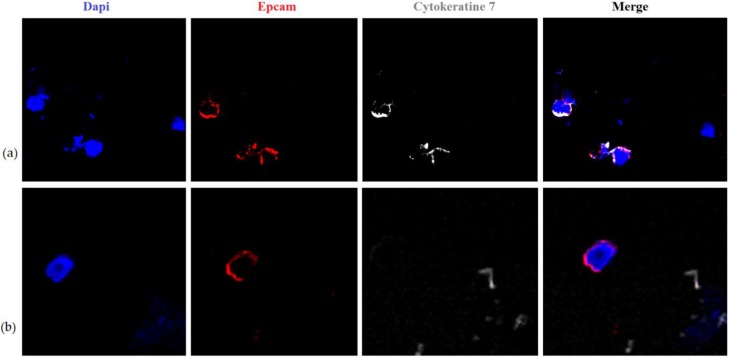
Characteristics of EpCAM-negative CTCs: (**a**) CTC-5 subset isolated from blood by EpCAM-CD45-CD44+CD24-Ncadherin- phenotype using the cell sorter MoFlo XDP (Beckman Coulter, Franklin Lakes, FL, USA). Lumpy EpCAM expression is observed in cytoplasm and in nuclei of tumor cells, CK7 is expressed in cytoplasm (confocal microscopy, ×630); (**b**) CTC-5 subset isolated from blood by EpCAM-CD45-CD44+CD24-Ncadherin+ phenotype using the cell sorter MoFlo XDP (Beckman Coulter, Franklin Lakes, FL, USA). Diffuse EpCAM expression is observed in cytoplasm of tumor cells, CK7 expression is absent (confocal microscopy, ×630).

For verification of the hypothesis, that loss of EpCAM membrane expression in stem CTCs (EpCAM-negative CTCs) is caused by EpCAM translocation into a cell and not by its true absence, we isolated CTC-5 (EpCAM-CD45-CD44+CD24-Ncadherin-) and CTC-6 (EpCAM-CD45-CD44+CD24-Ncadherin+) from blood using the cell-sorting technique. Further, using anti-EpCAM and anti-CK7 antibodies, we estimated the characteristics of intracellular EpCAM expression using confocal microscopy. Based on the results of this research, it was shown that, in the N-Cadherin-negative subset of stem cells CTC-5 (without EMT properties), both cytoplasmic and nuclear granular expression of EpCAM, combined with CK7 expression, were observed (Figure 1a). In N-Cadherin-positive stem cells CTC-6 (with EMT properties), only cytoplasmic diffuse expression of EpCAM was observed. Moreover, the expression of CK7 was absent (Figure 1b). It could be that the obtained results demonstrate the available information regarding the fact that EpCAM translocating into nuclei initiates EMT [21].

We suggest that localization of EpCAM expression in the CTC-5 and CTC-6 subsets show consecutive phases of the same process. In CTC-5, EpCAM is translocated into the cytoplasm and nuclei, where it may further initiate EMT. Furthermore, expression of CK7 (epithelial marker) remains while expression of N-Cadherin (mesenchymal marker) is absent. It seems that, in CTC-6, induction of EMT already occurred. There was no EpCAM expression in nuclei, CK7 expression was lost, but expression of N-Cadherin was still observed. It should be noted that rare cells with nuclear EpCAM expression were detected among CTC-5 and rare CK7-negative cells were detected among CTC-6. Apparently, the induction of EMT in stem CTCs by EpCAM translocation is not the only mechanism. In stem CTC-4, N-Cadherin expression is combined with EpCAM membrane expression.

Tumor cells in EMT are able to survive under genotoxic and other influences and are generally resistant to chemotherapy and radiotherapy [22]. It is known that cancer stem cells are also resistant to chemotherapy [23].

Stem and EMT properties are often combined. It was shown that, in RAS or HER2 overexpressing tumor cells, the subset with stem properties CD44+CD24- increased the potential of EMT [24,25]. Among breast cancer cells with the phenotype CD44+CD24-/low, there is a subtype with a “claudin-low” phenotype, which is thought to express many of the EMT-associated genes, such as FoxC2, Zeb, and N-Cadherin [26,27]. It is important to note that EMT inductors may cause the appearance of stem properties in cells [28,29]. The importance of EMT in the manifestation of different stages of metastases is well-acknowledged [30].

## 3. Materials and Methods

### 3.1. Research Materials

The prospective study includes 27 patients with invasive breast cancer T2-4N0-3M0 aged 32 to 60 years admitted for treatment in Cancer Research Institute, Tomsk National Research Medical Center. Neoadjuvant chemotherapy was carried out for 14 patients. 13 patients were in the group without NACT. Venous heparinized blood samples taken before and after biopsy, after each courses of chemotherapy on 3–7 day, and before surgical intervention have served as a material for the study. Venous blood samples (5 mL) were collected in tubes containing heparin and used in the analysis during 2 h. The study was approved by the Local Committee for Medical Ethics of our Institute, and informed consents were obtained from all patients prior to analysis. The clinicopathological parameters of the patients with breast cancer are presented in Table 3. Seven healthy volunteers were included in the control group.

### 3.2. Flow Cytometry

Different populations of CTCs were evaluated by flow cytometry using a BD FACSCanto II system (Becton, Dickinson and Company (BD), Franklin Lakes, FL, USA) with BD FACS Diva software. For this procedure, stabilized heparin venous blood was incubated with fluorochrome-labeled monoclonal antibodies to CD45 (clone F10-89-4, PE/Cy7) (Abcam, Cambridge, UK), CD44 (clone IM7, FITC) (Abcam, Cambridge, UK), CD24 (clone SN3, PE) (Abcam, Cambridge, UK), EpCAM (clone VU-1D9, PerCP/Cy5.5) (Abcam, Cambridge, UK) and CD325 (N-Cadherin) (clone 8C11, APC) (Biolegend, San Diego, CA, USA). Then, erythrocytes were lysed in lysis solution (BD FACS lysing solution) and washed twice with Cell Wash buffer. The cell pellet was resuspended in 1 mL of BD Flow buffer. All samples were stored in the dark at 4 °C and were analyzed within 1 h. Measurement was done against unstained control.

The following molecular markers for CTCs were analyzed: EpCAM, CD45, CD44, CD24 and CD325 (N-cadherin). The cells were then classified based on the evidence of the EMT and the presence or absence of stem cell markers: CTC-1 (EpCAM+CD45-CD44-CD24-Ncadherin-); CTC-2 (EpCAM+CD45-CD44-CD24-Ncadherin+); CTC-3 (EpCAM+CD45-CD44+CD24-Ncadherin-); CTC-4(EpCAM+CD45-CD44+CD24-Ncadherin+); CTC-5(EpCAM-CD45-CD44+CD24-Ncadherin-); CTC-6 (EpCAM-CD45-CD44+CD24-Ncadherin+).

The results are reported as the proportion of the number of events with respect to CD45 expression multiplied by the concentration of leucocytes in 1 μL of blood. To perform this analysis simultaneously with immunophenotyping, cells were placed in a hematology analyzerLH500 to determine the total number of blood leukocytes. The level of CTCs in the sample was expressed as the number of cells per μL of blood and was determined by the formula:

$$\mathrm{CTC},\;\mathrm{cells}\;\mathrm{per}\;\mu L\;=(\frac{\mathrm{Events}\;\mathrm{of}\;\mathrm{the}\;\mathrm{CTC}\;\mathrm{population}}{\mathrm{Events}\;\mathrm{of}\;\mathrm{CD}45+})\times \mathrm{Leukocyte}\;\mathrm{concentration},\;\mathrm{cells}\;\mathrm{per}\;\mu L$$

### 3.3. Fluorescence-Activated Cell Sorting

Stem EpCAM-negative CTCs (CTC-5 (EpCAM-CD45-CD44+CD24-Ncadh-) and CTC-6 (EpCAM-CD45-CD44+CD24-Ncadh+) subpopulations) were sorted using a cell sorter MoFlo XDP with Summit software (Beckman Coulter, USA). For this procedure, stabilized heparin venous blood was incubated with fluorochrome-labeled monoclonal antibodies to CD45 (PE/Cy7, mouse IgG1, clone HI30, BD Pharmingen, San Diego, CA, USA), CD44 (APC-H7, mouse IgG2b, clone G44-26, BD Pharmingen, USA), CD24 (PerCP-Cy5.5, mouse IgG2a, clone ML5, BD Pharmingen, USA), EpCAM (FITC, mouse IgG1, clone EBA-1, BD Pharmingen, USA) and CD325 (N-Cadherin) (PE, mouse IgG1, clone 8C11, BD Pharmingen, USA). Isotype control antibodies (mouse PE-Cy7 IgG1, mouse APC-H7 IgG2b, mouse PerCP-Cy5.5 IgG2a, mouse PE IgG1, BD Pharmingen, USA) were used for negative control. Then erythrocytes were lysed in 1 mL VersaLyse Lysing Solution (Beckman Coulter, USA). Side scatter and forward scatter profiles were used to eliminate cell doublets. Then EpCAM-CD45-CD44+CD24-Ncadh- and EpCAM-CD45-CD44+CD24-Ncadh+ cells were routinely sorted.

### 3.4. CTCs Spiking Experiment

Blood specimens (5 mL vacuum tubes with heparin) for three spiking experiments were drawn under informed consent from healthy donors and breast cancer patients at Cancer Research Institute, Tomsk National Research Medical Center according to a protocol approved by the Local Committee for Medical Ethics. Whole blood (100 μL) of healthy donor was spiked with the known number (range 18–33 CTCs) of CTCs which were isolated using a cell sorter MoFlo XDP with Summit software. Three samples of the enriched blood were counted on a flow cytometer. The results of the flow cytometry assay ranged from 89% to 103% (CV 6%).

### 3.5. Confocal Microscopy

Visualization of isolated subsets of stem EpCAM-negative CTCs was carried out on confocal microscope LSM 780 NLO (Carl Zeiss, On Cohen, Germany). In order to do that cell suspension after sorting was dried on glass with poly-l-lysin coating, fixated with cold methanol and incubated with 3% BSA in 1× PBS with 0.02% Tween 20 (Amresco, Dallas, TX, USA) during 45 min for preventing non-specific antibody binding. Further the primary antibodies rabbit anti-EpCAM (polyclonal, 1:2000, Abcam, Cambridge, UK) and goatanti-CK7 (polyclonal, 1:50, Santa Cruz Biotechnology, Dallas, TX, USA) in 1% BSA were added and incubated in dark during 30 min. Then were washed with PBS and after that the cocktail of second conjugated antibodies Anti-Rabbit IgG H&L (Cy3) (Abcam, Cambridge, UK) and Anti-Goat IgG H&L (AlexaFluor 647) (Abcam, Cambridge, UK) was added. Sections were covered by Mounting Medium with DAPI (Dako, Carpinteria, CA, USA) and analyzed with the laser scanning microscope LSM 780 NLO (Carl Zeiss, Oberkochen, Germany) with magnification ×630.

### 3.6. Statistical Analysis

All the statistical analyses were performed by using the Statistica 10.0. (data analysis software system) (StatSoft, Inc., Tulsa, OK, USA). The obtained data were processed using variation statistics. Assessment of the normal distribution of the results was performed using the Kolmogorov-Smirnov test. The significance of differences was assessed using the nonparametric Mann–Whitney test (for independent samples) and the Wilcoxon (Z) test (for dependent samples). Data are presented as the median (Me) and the upper and lower quartiles (Q1–Q3). Two-sided *p*-values of <0.05 were considered statistically significant.

## 4. Conclusions

Both EMT and stemness may be described by several markers. Thus, our data is true for cells with EMT and/or stemness markers used in this manuscript. Among the six studied CTC subsets, there were cells with stemness and EMT properties, with a combination of these properties, and also without any of them. CTC subsets analyzed in our study reflect both relatively stable cell phenotypes (e.g., cells without stemness and EMT and cells with stemness but without EMT features) and transient states which are peculiar to EMT. It can be stem cells that undergo EMT and display both the presence and absence of membrane expression of EpCAM as well as cytoplasmic/nuclear expression of EpCAM. In addition, non-stem cells in an EMT state with membrane expression of EpCAM and its loss can be also considered as transient cell states. Transient EMT phenotypes which are thought to be able to remain in fixed state for an indefinite time play a key role in invasion and metastasis as well as in chemotherapy resistance [31,32]. Epithelial-mesenchymal plasticity is believed to be maintained by several EMT programs. Moreover, the activation of EMT is probably a main mechanism of stem cell generation both from normal and carcinoma cells [31,33,34]. However, it is still unclear how EMT program is related to acquiring of stemness features [32]. Moreover, it is believed that EMT and stemness may occur independently from each other [35].

The quantity of tumor cells in peripheral blood resulted from three processes: Recruiting and intravasation in the tumor, destruction in remote organs, and extravasation in certain organs. In a heterogeneous population of CTCs, there are subsets of cells in which the quantity changed under the influence of NACT, as well as subsets of cells with no changes in terms of quantity. Increase of CTC-1 and CTC-6 quantity and a reduction of CTC-4 quantity after NACT do not eliminate its potential contribution in haematogenous metastases. The increase of CTCs might be evidence of preponderance of intravasation over intravascular death or extravasation, while the reduce of CTCs might be indicator of predominance extravasation over intravasation. It should be taken into account that, for haeamatogenous metastases development, the most important property of “cells-seeds” is the ability to effectively interact with cell elements and molecules of premetastatic niches [36,37].

It is reasonable to give special consideration to CTCs with a loss of EpCAM membrane expression and their intracellular translocation (CTC-5 and CTC-6). CTC subsets that are more likely responsible for metastases development are to be studied in further research. During NACT, it is necessary to consider the significant impact on heterogeneity of CTCs, which may increase the risk of haematogenous metastases.

## Figures and Tables

**Table 1:** Different populations of circulating tumor cells (CTCs) in the blood of breast cancer patients before biopsy, Me (Q1–Q3), cells per μL.

N	СTC Total	CTC-1	CTC-2	CTC-3	CTC-4	CTC-5	CTC-6
1		2	3	4	5	6	7
**Breast cancer patients**							
**27**	2.85 (0.51–4.40)	0.54 (0.00–1.11) *p*_2-1_ = 0.00003 *	0.09 (0.00–0.70) *p*_3-1_ = 0.00009 * p_3-2_ = 0.47 *	0.00 (0.00–0.43) *p*_4-1_ = 0.00002 * p_4-2_ = 0.0011 * p_4-3_ = 0.11 *	0.02 (0.00–0.63) *p*_5-1_ = 0.00009 * p_5-2_ = 0.33 * p_5-3_ = 0.43 * p_5-4_ = 0.68 *	0.18 (0.00–2.41) *p*_6-1_ = 0.00003 * p_6-2_ = 0.14 * p_6-3_ = 0.67 * p_6-4_ = 0.011 * p_6-5_ = 0.23 *	0.04 (0.00–0.34) *p*_7-1_ = 0.00006 * p_7-2_ = 0.57 * p_7-3_ = 0.47 * p_7-4_ = 0.79 * p_7-5_ = 0.58 * p_7-6_ = 0.19 *
**Healthy donors**							
**7**	0.00 (0.00–0.00)	0.00 (0.00–0.00)	0.00 (0.00–0.00)	0.00 (0.00–0.00)	0.00 (0.00–0.00)	0.00 (0.00–0.00)	0.00 (0.00–0.00)

* Wilcoxon Matched Pairs Test. Marked tests are significant at *p* < 0.05000.

**Table 2:** Effect of neoadjuvant chemotherapy on the level of different populations of CTCs in the blood of breast cancer patients Me (Q1–Q3), cells per μL.

Level after Biopsy	Level after 1 Course of NACT	Level after 2 Course of NACT	Level after 3 Course of NACT	Level before Surgical Treatment
1	2	3	4	5
**СTC total (EpCam+/-CD45-CD44+/-CD24-Ncadherin+/-)**				
2.97 (0.93–5.85)	2.51 (1.85–21.45) *p*_2-1_ = 0.32	11.04 (3.46–21.96) *p*_3-1_ = 0.77 *p*_3-2_ = 0.20	19.94 (9.28–130.86) *p*_4-1_ = 0.068 *p*_4-2_ = 0.26 *p*_4-3_ = 0.32	14.06 (4.51–74.60) *p*_5-1_ = 0.017 *p*_5-2_ = 0.035 *p*_5-3_ = 0.16 *p*_5-4_ = 0.77
**CTC-1 without stemness and EMT properties (EpCam+CD45-CD44-CD24-Ncadherin-)**				
0.90 (0.00–1.73)	1.25 (0.07–5.21) *p*_2-1_ = 0.074	3.75 (0.29–5.79) *p*_3-1_ = 0.027 *p*_3-2_ = 0.916	0.74 (0.31–4.80) *p*_4-1_ = 0.176 *p*_4-2_ = 0.735 *p*_4-3_ = 0.735	3.45 (0.00–7.09) *p*_5-1_ = 0.028 *p*_5-2_ = 0.600 *p*_5-3_ = 0.310 *p*_5-4_ = 0.310
**CTC-2 without stemness and with EMT properties (EpCam+CD45-CD44-CD24-Ncadherin+)**				
1.03 (0.45–22.50)	0.28 (0.17–0.66) *p*_2-1_ = 0.345	0.87 (0.39–5.99) *p*_3-1_ = 0.916 *p*_3-2_ = 0.345	0.83 (0.29–1.84) *p*_4-1_ = 0.463 *p*_4-2_ = 0.735 *p*_4-3_ = 0.600	0.28 (0.00–1.28) *p*_5-1_ = 0.865 *p*_5-2_ = 0.046 *p*_5-3_ = 0.753 *p*_5-4_ = 0.753
**CTC-3 with stemness and without EMT properties (EpCam+CD45-CD44+CD24-Ncadherin-)**				
0.02 (0.00–0.22)	0.12 (0.00–1.09) *p*_2-1_ = 0.310	0.20 (0.00–1.63) *p*_3-1_ = 0.176 *p*_3-2_ = 0.079	0.50 (0.00–1.79) *p*_4-1_ = 0.115 *p*_4-2_ = 0.115 *p*_4-3_ = 0.916	0.00 (0.00–2.57) *p*_5-1_ = 0.498 *p*_5-2_ = 0.224 *p*_5-3_ = 0.892 *p*_5-4_ = 0.753
**CTC-4 with stemness and EMT properties (EpCam+CD45-CD44+CD24-Ncadherin+)**				
0.22 (0.00–0.55)	0.00 (0.00–0.26) *p*_2-1_ = 0.715	0.05 (0.00–0.22) *p*_3-1_ = 0.345 *p*_3-2_ = 0.500	0.00 (0.00–1.64) *p*_4-1_ = 0.144 *p*_4-2_ = 0.108 *p*_4-3_ = 0.224	0.01 (0.00–0.71) *p*_5-1_ = 0.043 *p*_5-2_ = 0.043 *p*_5-3_ = 0.115 *p*_5-4_ = 0.892
**CTC-5 with stemness, without EMT properties and without EpCAM membrane expression (EpCam-CD45-CD44+CD24-Ncadherin-)**				
0.00 (0.00–0.49)	0.46 (0.00–2.61) *p*_2-1_ = 0.892	1.08 (0.13–5.32) *p*_3-1_ = 0.310 *p*_3-2_ = 0.310	5.62 (2.12–9.57) *p*_4-1_ = 0.068 *p*_4-2_ = 0.123 *p*_4-3_ = 0.025	2.39 (0.10–12.80) *p*_5-1_ = 0.086 *p*_5-2_ = 0.498 *p*_5-3_ = 0.128 *p*_5-4_ = 0.207
**CTC-6 with stemness and EMT properties and without EpCAM membrane expression (EpCam-CD45-CD44+CD24-Ncadherin+)**				
0.16 (0.04–1.59)	0.32 (0.06–0.87) *p*_2-1_ = 1.00	0.21 (0.02–2.35) *p*_3-1_ = 0.498 *p*_3-2_ = 0.865	2.95 (1.25–13.28) *p*_4-1_ = 0.027 *p*_4-2_ = 0.062 *p*_4-3_ = 0.310	3.19 (0.47–9.41) *p*_5-1_ = 0.027 *p*_5-2_ = 0.090 *p*_5-3_ = 0.345 *p*_5-4_ = 0.779

Wilcoxon Matched Pairs Test. Marked tests are significant at *p* < 0.05000.

**Table 3:** The clinicopathological parameters of the patients with breast cancer.

Clinicopathological Parameters	N (%)
**Age (year) (Me (Q1–Q3))**	
49 (43–57)	27 (100%)
**Molecular type of breast cancer**	
Luminal A	6/27 (22%)
Luminal В1	12/27 (44%)
Luminal В2	1/27 (4%)
HER2-positive	1/27 (4%)
Triple-negative	7/27 (26%)
**Tumor size**	
T1	6/27 (22%)
T2	18/27 (67%)
T3	1/27 (4%)
T4	2/27 (7%)
**Lymph node status**	
N0	14/27 (52%)
N1	8/27 (30%)
N2	3/27 (11%)
N3	2/27 (7%)
**Neoadjuvant chemotherapy (NACT)**	
NO	13/27 (48%)
YES	14/27 (52%)
